# Adult Triage in the Emergency Department: Introducing a Multi-Layer Triage System

**DOI:** 10.3390/healthcare13091070

**Published:** 2025-05-06

**Authors:** Dimitrios Tsiftsis, Andreas Tasioulis, Dimitrios Bampalis

**Affiliations:** 1 Emergency Department, Nikaia General Hospital, 184 54 Nikaia, Greece; adreastasioulis@gmail.com; 2 Emergency Department, General Hospital of Larisa, 413 34 Larisa, Greece; dbabales@yahoo.com

**Keywords:** triage, early warning scores, ESI, NEWS, HEART, emergency department

## Abstract

Emergency department (ED) triage is the cornerstone of ED operations. Many different triage systems have been proposed and implemented globally. To date, an ideal triage system has not yet been identified. As the burden on EDs rises, with overcrowding being recognized as a universal problem, ED triage needs to be restructured to address this reality. Extensive and critical literature research over the years has identified the strengths and weaknesses of current ED triage implementations. A novel multi-layer triage system was introduced and implemented in Greek Eds, combining the strengths of various triage and early warning systems and scores to minimize under-triage and the adverse downstream effects it creates on patient outcomes. Acknowledging that no triage system can be universally adapted in different settings, the structural concepts of this triage system address most of the triage problems currently reported in the literature.

## 1. Introduction

Emergency department (ED) overcrowding is a global phenomenon that delays diagnostic and therapeutic interventions [1]. This delay might crucially affect patient outcomes [2]. As ED patient volumes increase and even more people endure constantly prolonged waiting times, an accurate triage system becomes vital.

Hospital triage is a process through which healthcare professionals actively try to identify high-acuity patients and prioritize them accordingly. These patients range from critically ill, in need of immediate life-saving interventions, to patients with minor medical problems who are low urgency. The majority of the ED population lies between these two extremes. Both over-triage and under-triage hurt ED flow and patient waiting times. Under-triage might leave a critical patient in the waiting room for a prolonged period leading to severe deterioration. Over-triage will overflow the treatment area with lower acuity patients consuming all available treatment places and resources and thus preventing higher acuity patients from entering in a timely manner and prolonging their time in the waiting area.

Healthcare systems and emergency departments have developed triage systems that best fit their specific needs. Great variations exist not only between different healthcare systems but even among EDs in the same country regarding the triage model adopted, personnel performing triage, and the triage process itself. Whatever triage system is chosen, it must meet the following requirements of being useful, valid, and reproducible. It must also be easy to use and classify, to help medical staff determine the acuity level in the shortest possible time [1,3].

**Figure 1:**
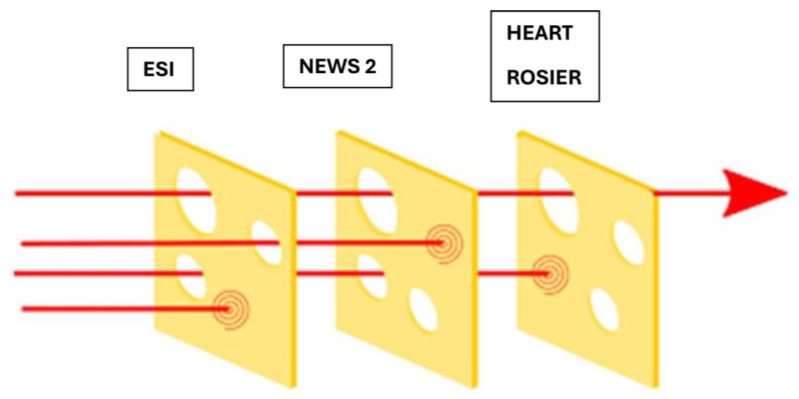
The multi-layer triage approach. Based on the Ben Aveling cheese model [6]. ESI, emergency severity index; NEWS 2, national early warning score 2.

The emergency department of Nikaia General Hospital, Nikaia, Greece is the busiest ED in Greece with more than 1000 ED visits in a 24 h shift. The emergency department of Larisa General Hospital, Greece has more than 300 ED visits in a 24 h shift. This overflow of incomings combined with other structural vulnerabilities of the Greek emergency healthcare system leads to prolonged waiting times from triage to seeing a physician with the right to treat, which in some cases may exceed 6 h. In 2018, we restructured our adult patient triage protocols, introducing a multi-layer triage approach based on the Swiss cheese model [4] (Figure 1). Our goal was to combine the strengths of accredited triage and early warning systems and scores to produce a process that would ensure that prolonged waiting times would not negatively impact patient outcomes [5].

Based on these principles, the Hellenic Society for Emergency Medicine developed a National Triage Proposal in 2024 [7].

## 2. Evaluating Existing Scoring Systems

Existing scoring systems can be roughly divided into three main categories.

### 2.1. Symptom-Based Triage Systems—Clinical Impression Triage Systems

The most widely used triage systems in this category are the Manchester triage system (MTS), the Australasian triage system (ATS), the emergency severity index (ESI), and the Canadian triage and acuity scale (CTAS). These 5-tier scoring systems are all well-validated and widely adopted in many EDs globally.

The outlines of each triage system are presented in Table 1.

There is still no clear advantage of one triage system over another [1,8,9].

Triage accuracy by all the above systems ranges between 56% and 87% [10]. All define very precisely and concordantly the highest (1 and 2) and the lowest (5) priorities while assigning priorities to the intermediate categories (3 and 4) was less precise for the adult population [1]. ESI has been reported to have a 20–30% under-tirage rate even for high acuity patients [11,12,13,14,15,16]. In our setting, when using ESI alone, accuracy was calculated at 63%, with an overall 23.6% under-triage rate [17]. Interpretation of vital sign deterioration has been identified as one of the factors leading to under-triage when using ESI [18].

The strengths of symptom-based triage systems are that they are validated, fast, simple, and reliable for higher acuity patients (Priority 1 and Priority 2) and very low acuity patients (Priority 5). Their weaknesses include a high percentage of under-triage even for high acuity patients. They remain mainly subjective (depending on the level of training of the triage personnel) and thus not reproducible.

We chose to implement the ESI triage system mainly because there are no preset response times for each triage category and it fits better with our practice and policies so far. By choosing ESI almost 80% of our high acuity patients and our very low acuity patients should be identified quickly and accurately. A second triage layer would be needed to find these under-triaged critically ill patients and sort out medium and low-acuity patients. Since ESI is not symptom-based, we added several critical presenting symptom clusters from MTS, ATS, and CTAS as “Red Flag” symptoms to be recognized and prioritized accordingly.

### 2.2. Early Warning Scores (EWSs)

Early warning scores are based on the concept that altered physiology often precedes patient deterioration and death. Derangements in simple physiological observations (vital signs) can identify patients at high risk of deterioration. By recording and grading multiple parameters simultaneously, subtle changes in vital signs can be used to initiate early emergency management [19,20]. Most widespread are the rapid acute physiology score (RAPS), modified EWS (MEWS), modified EWS with Glasgow coma scale (GCS) (MEWS GCS), rapid emergency medicine score (REMS), Goodacre score, Worthing physiological score (WPS), Groarke score, VitalPac EWS (ViEWS), abbreviated VitalPac EWS (AbViEWS), Glasgow coma scale-age-systolic blood pressure score, vital sign score (VSS), National EWS (NEWS), and vital sign group (VSG) scores.

Early warning scores can accurately predict outcomes in several different populations [21,22]. They are excellent predictors of cardiac arrest, ICU transfer, and death in ICU, mortality within 2 days, deterioration within 2 days, and hospital admissions [21,23]. Among their advantages are accuracy, cross-specialty application, impact on communication, and opportunity for automation [23]. Their weak points are sensitivity, especially compared to specialty-specific scores, the need for practitioner engagement, and the need for clinical judgment.

EWSs have been proposed as emergency department triage tools [22]. EWS triage outperforms symptom-based triage in high-acuity patient recognition and risk stratification of mid-acuity patients [24].

**Figure 2:**
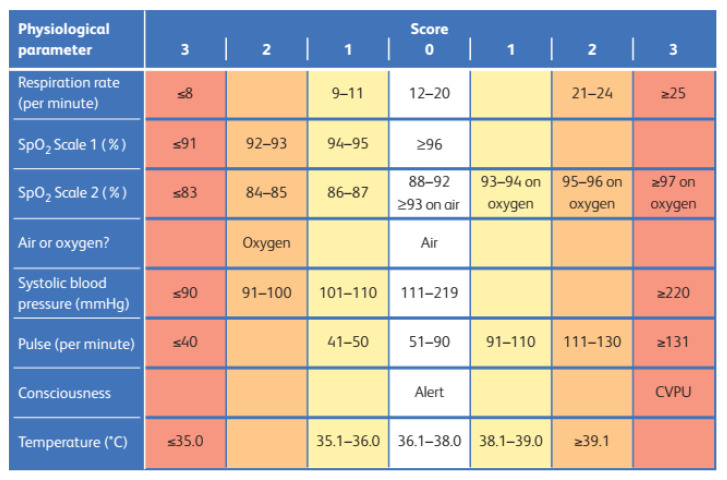
NEWS 2 chart. (Royal College of Physicians. National early warning score (NEWS) 2: standardizing the assessment of acute-illness severity in the NHS. Updated report of a working party. London: RCP, 2017).

**Figure 3:**
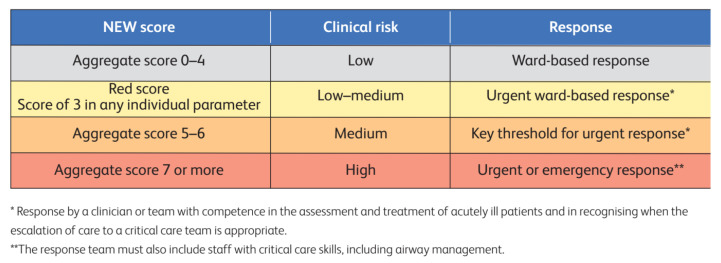
NEWS 2 thresholds and triggers. (Royal College of Physicians. National early warning score (NEWS) 2: standardizing the assessment of acute-illness severity in the NHS. Updated report of a working party. London: RCP, 2017).

There is no clear advantage to one EWS system over another [21]. We have chosen to integrate NEWS 2 into our multi-layer triage system. NEWS 2 is simple, easy to use, and reproducible. The vital signs are recorded on the table chart, and the score is calculated (Figure 2). Once the NEWS 2 score is calculated, appropriate response triggers are provided by the Royal College of Physicians (Figure 3) [25,26].

Summarizing. NEWS 2 is a validated and easy-to-use score [27]. By choosing to add NEWS 2 as a second layer in our triage process, we address vital signs interpretation which was one of the most common error points in the triage systems mentioned above. We decrease the probability of under-triaging high acuity patients as NEWS 2 has better sensitivity in detecting high acuity patients that have been under-triaged in the previous step. By using the integrated thresholds and triggers, patients can subjectively and reproducibly be further triaged as middle and low acuity. Adding this layer slightly prolongs the triage process. This time delay will have a minimal impact on patient outcomes as the Priority 1 and Priority 2 patients who need immediate treatment have already been transferred to the treatment area by using ESI.

### 2.3. Specific Disease Scores

Time-sensitive, high mortality conditions might have atypical [28] or confusing presentations [29,30] on arrival, and minimal vital signs deterioration. Both acute coronary syndromes (ACSs) and stokes are among the leading causes of death and disability and early recognition and timely intervention are critical. As stated earlier, EWSs underperform compared to specialty-specific scores [31]. To detect these patients that have slipped through the first two triage layers, an extra layer is added consisting of disease-specific scores.

Many scores are in use to help identify patients with these conditions promptly and accurately. The most commonly used to detect ACS are the thrombosis in myocardial infarction risk (TIMI) score, the global registry of acute coronary events (GRACEs) score, and the HEART score. Likewise, the National institutes of health stroke scale (ΝΙHSS), the Cincinnati prehospital stroke severity scale (CPSSS), the rapid arterial occlusion evaluation (RACE), the face arm speech test (FAST), the medic prehospital assessment for code stroke (MedPACS), and the recognition of stroke in the emergency room (ROSIER) are used for the early recognition of stroke.

**Figure 4:**
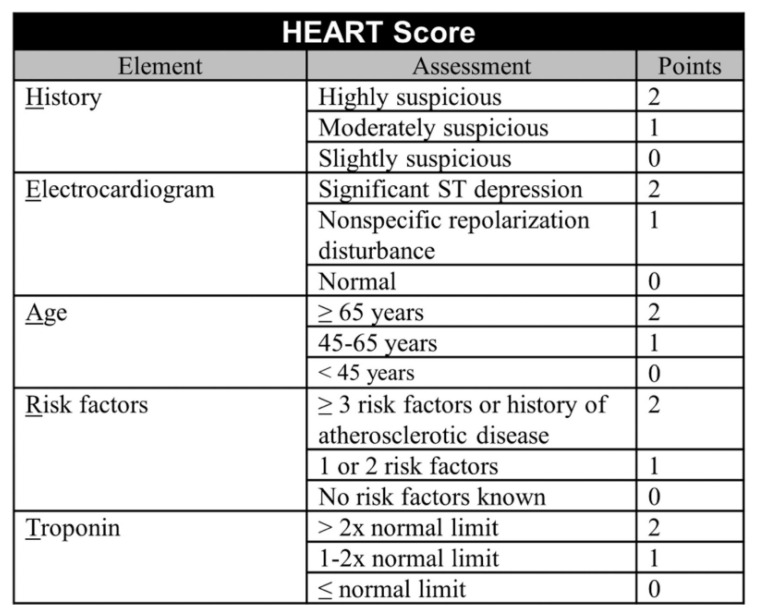
The HEART score [34].

**Figure 5:**
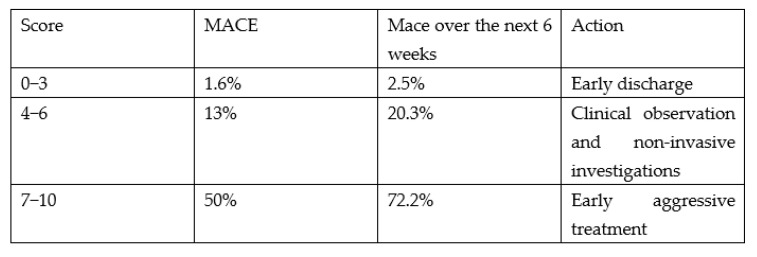
HEART score interpretation and stratification. (MACE = Major adverse cardiac events).

The HEART score seems to perform better than other scores in detecting ACS [32,33]. In the mnemonic HEART, each letter corresponds to one of the following key pieces of the evaluation for patients with chest pain: history, ECG, age, risk factors, and troponin. Each component is scored on a scale of 0–2, with total scores ranging between 0 and 10 (Figure 4). The calculated score corresponds to the short-term probability of a major adverse cardiovascular event (MACE) and appropriate action is recommended [6] (Figure 5).

**Figure 6:**
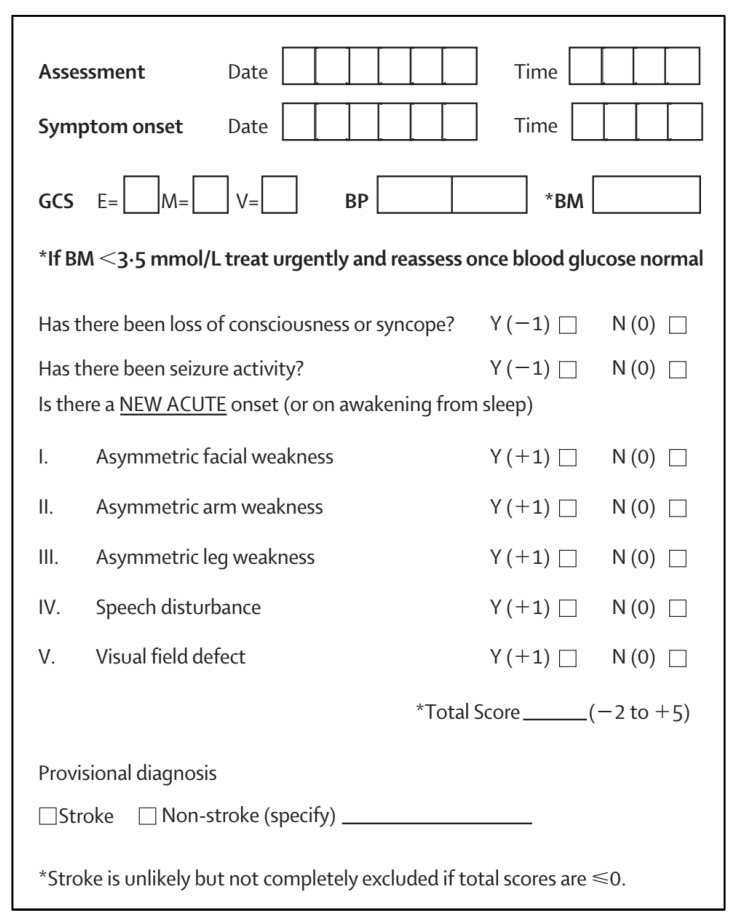
Rosier scored [37]. BM, blood glucose; BP, blood pressure (mm Hg); GCS, Glasgow coma scale; E = eye; M = motor; V = verbal component.

For detecting stroke in the emergency department, the ROSIER could be the test of choice as it has been well evaluated and showed consistently high sensitivity [35,36]. The ROSIER scale is a 7-item stroke recognition instrument employing clinical history and neurological signs, ranging from 2 to +5. A score of +1 or higher indicates a positive diagnosis of stroke or transient ischemic attack (TIA). The scale encompasses assessment criteria such as loss of consciousness, seizure activity, asymmetric facial, arm and leg weakness, speech disturbance, and visual field deficit (Figure 6).

Adding the ROSIER and HEART scores as an extra layer to our triage process minimizes the chance of under-triaging a life-threatening, time-sensitive disease. To perform these scores, certain blood tests are necessary. Blood glucose levels and troponin are essential parts of the algorithm. These create certain logistic needs (point care devices, blood sampling, personnel, etc.) that have a great impact on triage time. Depending on the setup, this might take from 10 to 30 min. In departments like ours with long waiting times, there is a clear benefit as under-triaged patients might lose their therapeutic window. The number of patients that end at this arm of our triage process is small and has minimal effect on the door-to-triage time for new incomings.

## 3. Proposing a Multilayer Triage System

**Figure 7:**
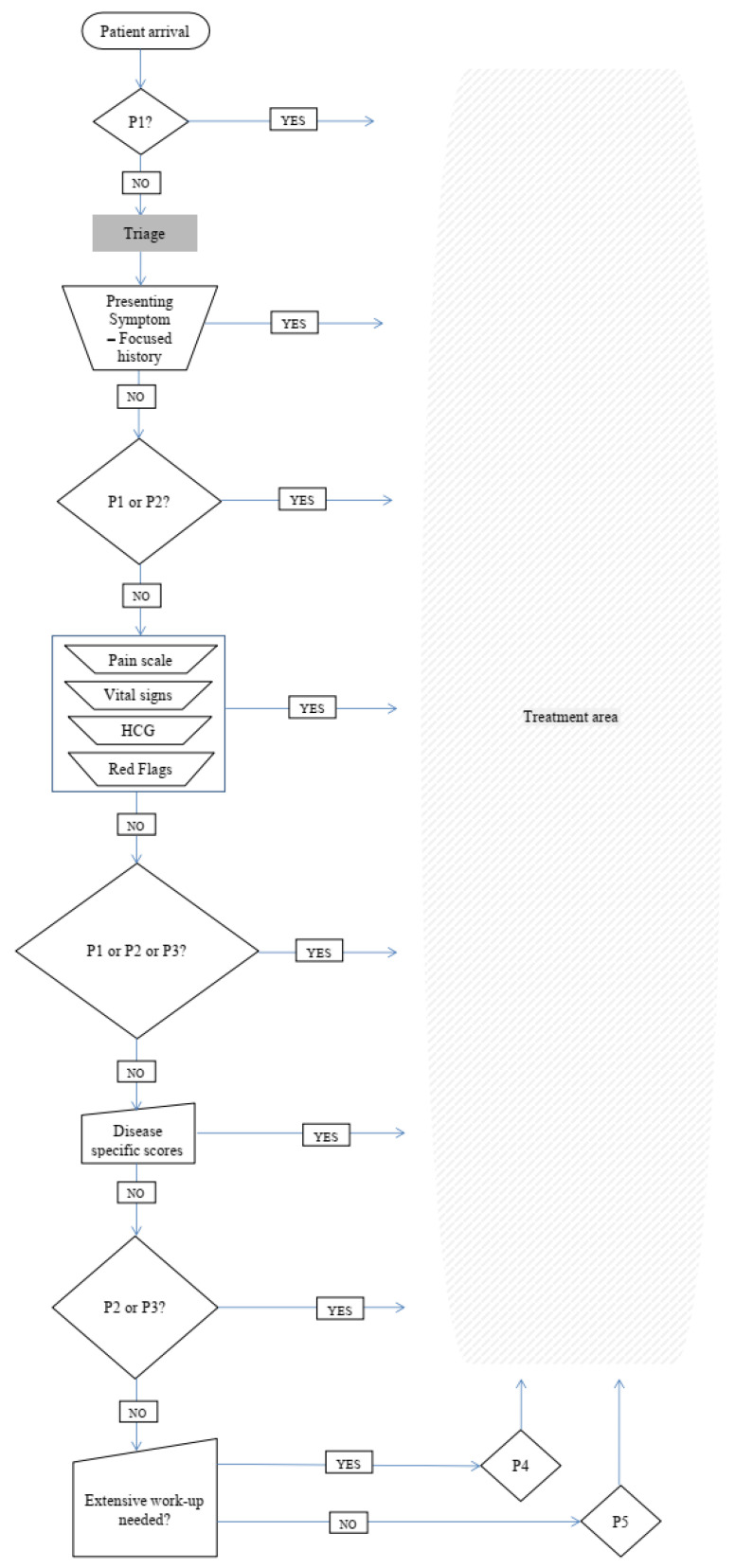
Scheme 1: the multilayer triage flow chart.

Having an in-depth understanding of our emergency department’s strengths and weaknesses and experience in conducting triage by ESI alone, we had identified areas of improvement of our triage process. Before introducing our institutional multilayer triage (Figure 7) system, an extensive and critical review of the literature was conducted. We combined the strong points of each score to better fit our needs. ESI would quickly and accurately identify the majority of very high acuity patients (Priority 1). ESI along with specific symptom clusters from ATS, MTS, and CTAS “Red flags” were used to identify quickly and accurately our high acuity patients (Priority 2). Acknowledging that almost 20% of Priority 1 and 2 patients might be under-triaged by ESI we introduced the NEWS 2 score as a second layer. In addition to increasing our Priority 1 and 2 detection rate, this addition also helped in a better interpretation of vital signs, and the objective and reproducible allocation of Priority 3 and Priority 4 patients. As an added benefit, it created an objective benchmark to which the patient’s improvement or deterioration over time and response to treatment could be compared. ACS and stroke detection were a priority and a third layer consisting of the ROSIER and HEART scores was added allocating patients to Priority 2, 3, or 4. Those patients who at the end of the triage process were characterized as having low acuity were prioritized as Priority 4 or Priority 5 according to the estimated extent of investigation needed according to combined elements of the ESI (resources) and CTAS (age and comorbidities). Table 2 summarizes our priority allocation tools and scheme 1 depicts our triage process.

The steps of the multilayer triage system are as follows:

- **Seeking Priority 1 patients**—Clinical impression.
  - Use the basic principles of clinical impression triage systems such as ESI;
  - “Is there an immediate risk for life or limb?”;
  - If the answer is YES, the patient is Priority 1;
  - If the answer is NO, proceed to the next step.
- **Seeking Priority 2 Patients**—Basic history taking and clinical impression.
  - Use the basic principles of clinical impression triage systems such as ESI;
  - “Is the patient’s condition serious enough or deteriorating so rapidly that there is the potential of threat to life or organ system failure?”;
  - “Is the patient in severe pain?”;
  - “Does the patient have altered mental status?”;
  - “Are there any “Red Flags”?” CTAS, ATS, and MTS;
  - If the answer is YES to any of the above questions, the Patient is Priority 2;
  - If the answer is NO, proceed to the next step.
- **Are you sure the Patient is NOT Priority 1 or 2?**—Vital signs.
  - Use NEWS 2 to interpret vital signs;
  - Prioritize the patient according to the EWS you have chosen;
    - NEWS2 Score > 7: the patient is Priority 2;
    - NEWS2 Score 5–6 or a red score of 3 in any individual parameter: the patient is Priority 3
    - NEWS2 Score 0–4: proceed to the next step.
- **Could the patient have an atypical presentation of a time-sensitive disease?**—Disease-specific scores.
  - Use one of the accredited disease-specific scores depending on the clinical question;
  - Prioritize the patient according to the score you have chosen;
  - Use the HEART score for a possible ACS;
    - For a HEART score of 7–10, the patient is Priority 2;
    - For a HEART score between 4 and 6, the patient is Priority 3;
    - For a HEART score of 0–3, proceed to the next step;
  - Use the Rosier score for a possible stroke;
    - For Rosier > 1, the patient is Priority 2;
    - For Rosier ≤ 0, proceed to the next step.
- **Will this patient require extensive work-up?**—Focused history-taking.
  - Use the basic principles of clinical impression triage systems such as ESI, and CTAS;
  - “Will the patient, due to his age or comorbidities, require extensive work-up?”;
  - If the answer is YES, the patient is Priority 4;
  - If the answer is NO, the Patient is Priority 5.

## 4. Results

Having implemented this multi-layer triage system for our adult population for more than 3 years, we have had almost no critical events in the waiting room. Few patients in the waiting room will need to change to a higher priority category while waiting. There is a very high level of agreement in the triage category between triage personnel of different training backgrounds. Although not systematically recorded, over-triage is below 15%. Our average triage time remains at 10 min. Even for the most complex arms of the chart, triage time never exceeds 25 min without prolonging our door-to-triage time. Our triage protocol has evolved over three years. A study protocol is running to evaluate its exact impact on triage accuracy and efficiency. A multi-center prospective study should be conducted to evaluate the effectiveness of this triage system in other EDs.

Having created an objective and reproducible system, training, reviewing, and quality control have become easier. Training triage healthcare personnel (ED medical doctors and nurses) is structured on our flowchart and the different scales used. Our training model consists of a 120 h theoretical course followed by 100 h supervised hands-on training. Monthly, our triage team critically reviews triage charts compared with patient outcomes and further didactic interventions are scheduled where needed.

More recently we have created and started implementing an artificial intelligence decision assistance tool built on these parameters. Artificial intelligence in triage has recently been a field of intensive research. Although the first reports seem very promising, there is still a lot to be undertaken until AI is widely available for ED triage [38,39].

Re-structuring the triage process alone has had multiple downstream effects with a positive impact on triage accuracy. A structured training program was implemented for triage personnel. Constant and systematic training of triage personnel has been shown to have a clear impact on triage quality. Our triage training program is being gradually adopted across Greece. Due to the new triage system, charting and recording had to be re-structured. Complete charting and strict adherence to the triage process have been identified as major contributors to high-quality triage. Re-triage at set time intervals was introduced as part of the quality control of the multilayer triage system. This created a re-triage culture that has remained. Even during our initial needs assessment and planning of the triage system, several ED throughput issues were identified and addressed accordingly.

## 5. Conclusions

Triage is the most vital part of ED operations and can have a great impact on ED flow and patient outcomes. The perfect triage system does not exist. Comparing different triage models is very difficult as this is a multi-factorial process depending on the input and output of patients, ED resources, staffing and hospital capabilities, training background of triage personnel, etc. Each emergency department must identify its own needs and tailor a triage process to fulfill them.

## Figures and Tables

**Table 1:** Characteristics of the most important five-level triage systems.

Parameter	ATS	MTS	CTAS	ESI
Time to initial assessment	10 min	ns	ns	ns
Time to contact with doctor with the right to treat	Immediate/10/60/120 min	Immediate/10/60/120/240 min	Immediate/15/30/60/120 min	Immediate/10 min/n. s
Re-triage	ns	As required	I:continuously; II: 15 min; III: 30 min; IV: 60 min; V: 120 min	As required
List of diagnoses or key symptoms	YES	52 key symptoms	YES	No
Training material	YES	YES	YES	YES

ATS, Australasian triage scale (previously national triage scale, NTS); CTAS, Canadian triage and acuity scale; MTS, Manchester triage scale; ESI, emergency severity index; ns, not specified; I to V: triage priority levels.

**Table 2:** Priority allocation summary.

Patient Priority	Clinical Condition	Tools Used to Identify
Priority 1	Immediate risk for life or limb	ESI
Priority 2	Serious enough or deteriorating so rapidly	ESI **and/or** Red Flags **and/or** NEWS 2 > 7 **and/or** HEART score >7 **and/or** Rosier > 1
Priority 3	Not serious enough, but could have atypical or early presentation of a serious condition	NEWS 2 = 5–6 **and/or** HEART score = 4–6
Priority 4	No serious underlying condition, but will require extensive work-up	ESI **and** NEWS 2 = 0–4 **and** HEART = 0–3 **and** Rosier ≤ 0
Priority 5	Acute but non-urgent or chronic problem without deterioration. Needs minimum investigation	ESI **and** NEWS 2 = 0–4 **and** HEART = 0–3 **and** Rosier < 0

## Data Availability

No new data were created or analyzed in this study.
